# Increased FGF23 serum level is associated with unstable carotid plaque in type 2 diabetic subjects with internal carotid stenosis

**DOI:** 10.1186/s12933-015-0301-5

**Published:** 2015-10-12

**Authors:** Federico Biscetti, Giuseppe Straface, Carlo Filippo Porreca, Giovanni Bertoletti, Claudio Vincenzoni, Francesco Snider, Egidio Stigliano, Vincenzo Arena, Flavia Angelini, Giovanni Pecorini, Antonio Bianchi, Raffaele Landolfi, Andrea Flex

**Affiliations:** Institute of Rheumatology and Affine Sciences, Catholic University School of Medicine, Rome, Italy; Laboratory of Vascular Biology and Genetics, Catholic University School of Medicine, Rome, Italy; Department of Medicine, A. Gemelli University Hospital, Catholic University School of Medicine, Rome, Italy; Department of Medicine, St M. Goretti Hospital, Latina, Italy; Department of Vascular Surgery, St M. Goretti Hospital, Latina, Italy; Department of Vascular Surgery, A. Gemelli University Hospital, Catholic University School of Medicine, Rome, Italy; Department of Pathology, A. Gemelli University Hospital, Catholic University School of Medicine, Rome, Italy; Department of Endocrinology, A. Gemelli University Hospital, Catholic University School of Medicine, Rome, Italy

**Keywords:** Type 2 diabetes, FGF23, OPG, Internal carotid artery stenosis, Unstable plaque

## Abstract

**Background:**

The object of this study was to investigate the potential role of FGF23 on plaque stability in type 2 diabetic patients with internal carotid artery stenosis.

**Methods:**

In this retrospective observational study, we analyzed FGF23 serum level in 361 type 2 diabetic patients with internal carotid artery stenosis undergoing carotid endarterectomy and in 598 diabetic controls without carotid atherosclerosis.

**Results:**

We found that FGF23 median serum levels was significantly higher in patients than in diabetic controls [67.7 (59.5–77.8) pg/mL and 43.89 (37.5–50.4), P < 0.001] and was significantly and independently associated with unstable plaque in patients with internal carotid artery stenosis [OR, 5,71 (95 % CI, 2.09–15.29].

**Conclusions:**

We have found, for the first time, that FGF23 could be associated with unstable plaque in type 2 diabetic patients with internal carotid artery stenosis.

## Background

Cerebrovascular diseases are a major cause of death and several studies found that unstable plaques increased the risk of ischemic stroke [1]. An important role in the mechanism of mineral metabolism, fundamental in plaque stability, is played by glycoprotein fibroblast growth factor 23 (FGF23) [2, 3]. FGF23, a hormone secreted primarily by osteocytes and to a lesser extent by osteoblasts, is involved in the regulation of vitamin D metabolism, phosphorus homeostasis and bone mineralization. In particular, it inhibits activation of calcitriol [1,25(OH)_2_D], induces urinary phosphorous excretion and suppresses parathyroid hormone (PTH) synthesis [3–5]. In patients undergoing hemodialysis, increased serum FGF23 levels were an independent risk of coronary artery disease (CAD) and mortality in patients with chronic kidney disease [6, 7]. Recently, FGF23 has been found to be associated with vascular dysfunction and total body atherosclerosis [8, 9]. Hu et al. have shown that serum FGF23 levels exhibit positive and independent associations with the presence of CAD and the cumulative number of stenotic vessels [10]. On the other hand, in another study on patients with normal renal function, the authors report that circulating FGF23 did not correlate with coronary artery calcification [11]. Increased FGF23 levels, in patients with cardiovascular disease, were a predictor of cardiac events [12] and have been associated with left ventricular hypertrophy [9, 13] and prevalent cardiovascular disease in elderly individuals with normal renal function in the population studied [14]. Despite the potential involvement of FGF23 in vascular calcification, clinical data on the association between serum FGF23 and cardiovascular pathology are limited and discordant [6, 15]. It is not completely understood how the association between FGF23 concentrations and outcome is influenced by the background cardiovascular risk and might be different between population-based cohorts compared to those selected for elevated cardiovascular risk or previous events. Type 2 diabetes (T2D) has been associated with higher rates of cardiovascular disease, thus showing the evaluation of FGF23 and its relationship with cardiovascular disease in T2D is of interest. Although the role of FGF23 in the development of atherosclerosis is suggested, there has been no clinical evidence about FGF23 levels and carotid plaque stability. The present study evaluated whether serum FGF23 level is associated with internal carotid artery stenosis, defined as high-grade (>70 %) carotid stenosis (ICAS), and with carotid plaque stability and correlates with other inflammatory cytokines [High-Sensitivity C-reactive protein (Hs-CRP) and interleukin-6 (IL-6)]. We also included osteoprotegerin (OPG) levels in analyzing the possible association of FGF23 with unstable plaque.

## Methods

### Study Population

We studied 361 consecutive T2D patients, with ICAS (mean age 72.3 ± 4.2 years) who underwent a carotid endarterectomy, recruited among subjects consecutively admitted to the Department of Vascular Surgery at the A. Gemelli University Hospital of Rome and to the Department of Vascular Surgery at the St M. Goretti Hospital, Latina (Italy) and 598 diabetic subjects (mean age 71.9 ± 3.9 years) without internal carotid artery stenosis (WICAS), recruited among subjects consecutively admitted to the Department of Internal Medicine at the A. Gemelli University Hospital of Rome from 1 January 2009 to 15 February 2015. The diabetic patients WICAS were matched according to sex and age and were without Doppler ultrasound evidence of atherosclerosis or clinical or radiologic evidence of cerebrovascular disease. A carotid endarterectomy was performed according to established criteria [16–18]. All subjects were Caucasian, from central and southern Italy. All participants had undergone a basic vascular evaluation, including a clinical vascular examination, a thorough color-coded echo flow imaging of the accessible arterial tree and an ECG at rest. The patients underwent an additional neurological evaluation and a cerebral CT to assess symptoms and/or cerebral infarction related to ICAS. For each study patient, clinical data and history regarding risk factors such as age, hypertension, hypercholesterolemia and smoking were obtained. Height and weight were measured and the body mass index (BMI) calculated as weight/height^2^ (kg/m^2^). Diabetes mellitus was determined by the presence of an existing diagnosis, fasting blood glucose > 126 mg/dL, glycohemoglobin A1c >5.8 % or by use of anti-diabetic medication or insulin. Hypertension was defined as a systolic blood pressure >130 mmHg and a diastolic blood pressure > 85 mmHg or by use of antihypertensive medications. Hypercholesterolemia was determined by a serum cholesterol value of >220 mg/dL or by use of cholesterol-lowering medications. Patients were classified as nonsmokers, if they had never smoked or if they had stopped smoking ≥1 year before the study. All other patients were classified as smokers. We calculated the estimated glomerular filtration rate (eGFR) with the Chronic Kidney Disease Epidemiology Collaboration equation (eGFR = 175 × standardized S_creatinine_^−1.154^ × age^−0.203^ × 1.212 [if black] × 0.742 [if female]), in which the GFR is expressed as mL/min per 1.73 m^2^ of body surface area and S_creatinine_ is expressed in mg/dL [6]. The eGFR was categorized according to established clinical cutoff points: ≥90, 60–89 and 15–59 mL/min per 1.73 m^2^. No participants in our sample had an eGFR <60 ml/min.

The preoperative evaluation included an ultrasound assessment of plaque density by color-coded echo flow imaging confirmed by angiography. Patients with malignant neoplasms, severe renal (eGFR <60 ml/min) or liver disease, serous membrane chamber fluid, severe oedema, hypothyroidism and osteoporosis were also excluded from the study. No patient was taking any drugs, such as oestrogen supplements, thyroxine, glucocorticoid, immunosuppressive, biphosphonate and warfarin. Approval for this study was provided by the ethics committees of the A. Gemelli University Hospital of Rome and St M. Goretti Hospital, Latina (Italy). Informed consent was obtained from participating patients.

### Histological assays

After a carotid endarterectomy, the specimens were briefly rinsed in normal saline solution and then immersed in a buffered 10 % formalin fixative and, subsequently, in a decalcifying solution (formic acid). The plaques were partly decalcified in order to be sectioned. Each specimen was sectioned transversely, perpendicular to the lumen, into 4 mm blocks, starting from the specimen base, and then, progressing distally until the whole specimen, including the bifurcation, was cut. Each block was processed in paraffin, cut into 4 μm sections, and then, the proximal end of one slice per block was stained in sequence with hematoxylin and eosin 1 % (H&E). The sections were examined for the presence of atheroma, a necrotic core, hemorrhage, fibrosis, calcification and thrombosis. Minimum magnification was used to evaluate the relative necrotic core, whereas, maximum magnification was used to evaluate the presence of hemorrhaging and calcification. The morphological study focused on the level of the largest plaque area, which frequently used to correspond to the level of maximal stenosis. The necrotic core was usually located in the deeper regions of the plaque and consisted of cholesterol clefts and amorphous material without any viable cells or admixed collagen. Calcifications were manifested as dark blue, sharply demarcated regions devoid of cells in the H&E stains. Intra-plaque hemorrhaging appeared as debris containing degenerated red blood cells, as well as macrophage engulfment of hemosiderin and giant cell development. Carotid plaques were classified as stable or unstable according to the American Heart Association (AHA) criteria, as defined by Stary and modified by Virmani [19, 20].

### Biochemical investigation

White blood cell count, serum creatinine, fasting cholesterol, triglycerides and low-density lipoprotein were measured. Blood samples were collected from all the subjects after an overnight fast. Serum was separated by centrifugation of blood samples, stored at −80°C until assayed and were treated the same way. Plasma FGF23 levels were measured using a second-generation C-terminal human enzyme-linked immunosorbent assay (Immutopics, San Clemente, CA). The coefficient of variation was 9.8 %. HsCRP was determined by using a high-sensitivity ELISA kit (Biocheck Laboratories, Toledo, OH). A monoclonal mouse antihuman OPG antibody was used as a capture antibody and a biotinylated polyclonal goat anti-human OPG antibody was used for detection. The intra and inter-assay coefficients of variation were 3.6 and 10.6 %, respectively. The sensitivity, defined as the mean ± 3 SD of the 0 standard, was calculated to be 0.15 pmol/ml. IL-6 levels were assessed by using the Quantikine ELISA kit (R&D systems, Minneapolis, MN). The serum levels were measured twice in each participant and the results were averaged.

### Statistical analysis

Demographic and clinical data between the groups were compared by a Chi squared test and by a *t* test. FGF23, HsCRP, OPG and IL-6 serum levels were compared by the Mann–Whitney test. Using a multivariate stepwise logistic regression analysis, two models were tested. The first one adjusted for conventional risk factors, while in the second model, FGF23 and OPG were included for testing. All analyses were performed by using the STATA version 11.0 for Windows (Statistics/Data Analysis, Stata Corporation, College Station, Texas, USA). Statistical significance was established at P < 0.05.

## Results

The demographic and clinical data of patients are shown in Table 1. There were no significant differences between the groups in terms of sex (*p* = 0.561), age (*p* = 0.204), diabetes duration (*p* = 0.247), smoking current and former (*p* = 0.145 and *p* = 0.112 respectively), phoshate and calcium levels (*p* = 0.234 and 0.763 respectively). In contrast, the body mass index (BMI), hypertension, coronary artery disease (CAD), history of ischemic stroke (HIS), peripheral artery disease (PAD), hypercholesterolemia were significantly more frequent in patients than controls (Table 1).

**Table 1:** Demographic and clinical data of study participants

	ICAS	WICAS	P value
	(n = 361)	(n = 598)	
Men/female, n	125:236	228:370	0.561^†^
Age (years ± SD)	72.3 ± 4.2	71.9 ± 3.9	0.204*
Diabetes duration, (years ± SD)	13.1 ± 4.2	12.4 ± 3.5	0.247*
BMI (Kg/m^2^ ± SD)	30.2 ± 6.5	27.6 ± 4.8	<0.01*
Smoking (current), n (%)	125 (34.6)	194 (32.4)	0.145^†^
Smoking (former), n (%)	163 (45.2)	279 (46.7)	0.112^†^
Hypertension, n (%)	234 (64.8)	248 (41.5)	<0.01^†^
CAD, n (%)	201 (55.7)	191 (31.9)	<0.01^†^
HIS, n (%)	174 (48.2)	0 (0.0)	<0.01^†^
PAD, n (%)	115 (31.9)	106 (17.7)	<0.01^†^
Hypercholesterolemia, n (%)	291 (80.6)	161 (26.9)	<0.01^†^
LDL-C (mg/dL ± SD)	213 ± 29.8	149 ± 21.2	<0.01^†^
Triglycerides (mg/dL ± SD)	184 ± 21.3	143 ± 18.8	<0.01^†^
Statins, n (%)	295 (81.7)	165 (27.6 %)	<0.01^†^
Antihypertensive drugs, n (%)	229 (63.4)	237 (39.6)	<0.01^†^
Anti-diabetic treatment			
Diet only, n (%)	38 (10.5)	65 (10.9)	0.563^†^
Oral Agents, n (%)	178 (49.3)	292 (48.8)	0.428^†^
Insulin therapy, n (%)	145 (40.2)	241 (40.3)	0.394^†^
eGFR (mL/min/1.73 m^2^ ± SD)	68.8 ± 15.2	91.1 ± 19.8	<0.001
Phosphate (mg/dL ± SD)	3.01 ± 1.32	2.89 ± 1.13	0.234
Calcium, mg/dL	9.3 ± 0.4	9.5 ± 0.6	0.763

*BMI* body mass index, *CAD* coronary artery disease, *HIS* history of ischemic stroke, *PAD* peripheral artery disease, *LDL-C* low-density lipoprotein cholesterol, *eGFR* estimated glomerular filtration rate

* Statistical test performed with Student’s t-test

^†^Chi square test for categorical values

Interestingly, FGF23, HsCRP, OPG and IL-6 median serum levels, were significantly higher in patients than in control subjects (Table 2). In particular the median serum FGF23 was 67.7 (59.5–77.8) pg/mL in ICAS and 43.89 (37.5–50.4) pg/mL in controls (*p* < 0.001), the median serum HsCRP was 7.95 (6.48-9.55) mg/L in ICAS and 3.95 (2.34–5.31) mg/L in controls (*p* < 0.001), the median serum OPG was 4.84 (3.52–5.95) pmol/L in ICAS and 2.48 (1.69–3.35) pmol/L in controls (*p* < 0.001) and the median serum IL-6 was 62.3 (57.1–68.5) pg/mL in diabetic patients with ICAS and 39.1 (33.8–45.2) pg/mL in diabetic controls (*p* < 0.001) (Table 2).

**Table 2:** Serum levels in diabetic patients with and without ICAS

Variables	ICAS	WICAS	P value
	(n = 361)	(n = 598)	
FGF23, pg/mL (IQR)	67.7 (59.5–77.8)^‡^	43.89 (37.5–50.4)^‡^	<0.001*
HsCRP, mg/L (IQR)	7.95 (6.48–9.55)^‡^	3.95 (2.34–5.31)^‡^	<0.001*
OPG, pmol/L (IQR)	4.84 (3.52–5.95)^‡^	2.48 (1.69–3.35)^‡^	<0.001*
IL-6, pg/mL (IQR)	62.3 (57.1–68.5)^‡^	39.1 (33.8–45.2)^‡^	<0.001*

*FGF23* fibroblast growth factor-23, *HsCRP* high-sensitivity C-reactive protein, *OPG* osteoprotegerin, *IL-6* interleukin-6

* χ2 test for categorical values

^‡^Median (interquartile range)

Subsequently, we divided the 361 diabetic patients with ICAS into unstable (n = 166) (USP) and stable (n = 195) plaque (SP) groups. The parameters analyzed in these patients are shown in Table 3. Interestingly, hypertension, HIS, hypercholesterolemia and low-density lipoprotein cholesterol (LDL-C) were significantly more frequent in patients with USP than in those with SP (Table 3). Table 4 analyzed FGF23, HsCRP, OPG and IL-6 median serum levels in patients with USP and SP. We found that the median levels of the these cytokines were significantly higher in USP than in SP subjects [FGF23, 78.4 (68.4–87.5) vs 34.7 (29.7–41.1) pg/mL, *p* < 0.001; HsCRP, 9.31 (7.94–11.1) vs 2.74 (1.92–4.12) mg/L, *p* < 0.001; OPG, 6.04 (4.65–7.34) vs 2.12 (1.02–2.95) pmol/L, *p* < 0.001; IL–6, 71.5 (66.3–77.4) vs 32.6 (28.8–36.6) pg/mL, *p* < 0.001] (Table 4).

**Table 3:** Demographic and clinical data of ICAS patients with USP and SP

	USP	SP	P value
	(n = 166)	(n = 195)	
Men/female, n	59:107	71:124	0.372^†^
Age (years ± SD)	72.1 ± 3.9	72.3 ± 4.1	0.315*
Smoking (current), n (%)	58 (34.9)	67 (34.4)	0.274^†^
Smoking (former), n (%)	74 (44,6)	89 (45.6)	0.112^†^
Hypertension, n (%)	119 (71.7)	115 (60.0)	0.004^†^
CAD, n (%)	92 (55.4)	109 (55.9)	0.159^†^
HIS, n (%)	102 (61.4)	72 (36.9)	0.001^†^
PAD, n (%)	53 (31.9)	62 (31.8)	0.195^†^
Hypercholesterolemia, n (%)	141 (85.0)	150 (76.9)	0.003^†^
LDL-C (mg/dL ± SD)	220 ± 31.4	206 ± 27.4	0.03^†^
eGFR (mL/min/1.73 m^2^ ± SD)	67.3 ± 13.1	69.1 ± 12.7	0.593

*CAD* coronary artery disease, *HIS* history of ischemic stroke, *PAD* peripheral artery disease, *LDL-C* low-density lipoprotein cholesterol, *eGFR* estimated glomerular filtration rate

* Statistical test performed with Student’s t-test

^†^Chi square test for categorical values

**Table 4:** Serum levels in diabetic patients with unstable and stable plaques

	USP	SP	
	(n = 166)	(n = 195)	
FGF23, pg/mL (IQR)	78.4 (68.4–87.5)^‡^	34.7 (29.7–41.1)^‡^	<0.001*
HsCRP, mg/L (IQR)	9.31 (7.94–11.1)^‡^	2.74 (1.92–4.12)^‡^	<0.001*
OPG, pmol/L (IQR)	6.04 (4.65–7.34)^‡^	2.12 (1.02–2.95)^‡^	<0.001*
IL-6, pg/mL (IQR)	71.5 (66.3–77.4)^‡^	32.6 (28.8–36.6)^‡^	<0.001*

*FGF23* fibroblast growth factor-23, *HsCRP* high-sensitivity C-reactive protein, *OPG* osteoprotegerin, *IL-6* interleukin-6

* χ^2^ test for categorical values

^‡^Median (interquartile range)

Finally, a multivariable stepwise logistic regression analysis revealed that, in model 1, making adjustments for traditional cardiovascular risk factors and established inflammatory cytokines, sex, age, smoking, hypertension, hypercholesterolemia, triglycerides, LDL-C, HsCRP and IL-6 levels were independent determinants of USP in T2D patients with ICAS. When FGF23 and OPG were included in the multivariable analysis (model 2), FGF23 and OPG remained independently associated with USP in ICAS patients, and the majority of the conventional risk factors in model 1 persisted in being determinants of ICAS in model 2 (Table 5).

**Table 5:** Multivariable stepwise logistic regression model for presence of USP

	Variable OR (95 % CI)	P value
Model 1		
Sex	1.03 (0.98–1.10)	0.110
Age	1.19 (1.13–1.29)	0.229
Smoking (current)	26.33 (4.24–181.32)	0.001
Smoking (former)	1.15 (1.06–1.23)	0.142
Hypertension	19.23 (4.879–86.12)	<0.001
Hypercholesterolemia	6.45 (1.57–26.97)	0.019
Triglycerides	0.45 (0.11–1.03)	0.035
LDL-C	9.45 (1.39–86.25)	0.039
HsCRP	35.03 (8.16–139.03)	<0.001
IL-6	5.51 (2.05–16.39)	0.002
Model 2		
Sex	0.85 (0.20–0.96)	0.083
Age	1.39 (1.07–1.55)	0.169
Smoking (current)	65.8 (3.91–880.7)	0.002
Smoking (former)	1.22 (1.11–1.28)	0.175
Hypertension	32.05 (5.11–195.56)	<0.001
Hypercholesterolemia	8.97 (1.14–48.37)	0.016
Triglycerides	0.85 (0.34–1.44)	0.004
LDL-C	18.24 (2.31–183.58)	0.017
HsCRP	73.79 (9.69–556.45)	<0.001
IL-6	8.69 (2.89–19.79)	<0.001
OPG	3.93 (1.81–5.37)	0.039
FGF23	5.89 (2.15–15.45)	0.009

## Discussion

Our present study is the first report to show that high serum levels FGF23 are independently associated with unstable carotid plaque in the general population with T2D. Our findings are consistent with previous reports that have found increased levels of FGF23 are an independent risk factor for adverse cardiovascular events [9].

In our study we found that the FGF23 median serum levels were significantly higher in T2D patients with ICAS than in diabetic control [67.7 (59.5–77.8) pg/mL and 43.89 (37.5–50.4) pg/mL (*p* < 0.001) respectively]. In addition, after all 361 diabetic patients with ICAS had been divided into unstable and stable plaque, we found that the FGF23 median levels of the same cytokines were significantly higher in USP than in SP subjects [78.4 (68.4–87.5) vs 34.7 (29.7–41.1) pg/mL, *p* < 0.001]. In our current study, we confirmed our previous report in which OPG serum levels were higher in patients with USP than in SP. We found that variant genotypes of the OPG gene and high serum OPG as an independent risk factor may contribute to the pathogenesis of carotid atherosclerosis. The associations between OPG and carotid plaque demonstrated in this study support further investigation into the effects of OPG on plaque stability and the possible role of OPG as a biomarker to identify patients with, or at risk of, cerebrovascular events [21].

In the final step, a multivariable stepwise logistic regression analysis revealed FGF23 and OPG remained independently associated with USP in ICAS diabetic patients (model 2) and the majority of the conventional risk factors in model 1 persisted in being determinants of ICAS in model 2.

In the pathogenesis of atherosclerosis, the potential role of FGF23 may be partially explained through its involvement in the complex process of vascular calcification. In the atherosclerotic process, vascular calcification plays an important role and the rupture of unstable plaques leads to critical atherosclerotic stenosis and acute cerebrovascular or cardiovascular syndrome [22]. Several studies showed that higher FGF23 levels were associated with the development of artery calcification, particularly in the presence of chronic kidney disease [23, 24]. Recently Masai et al. found serum FGF23 levels were also associated with coronary calcification independent of classical cardiovascular risk factors in patients with suspected CAD and with preserved renal function [9], while Xiao et al. reported an independent association between circulating FGF23 concentration and the severity and extent of coronary artery stenosis in the coronary angiographic patients [25]. Thus, it is reasonable to think FGF23 could play an important role in the vascular calcification process, since vascular calcification is associated with atherosclerosis, which may help explain the association between elevated FGF23 and the severity of atherosclerotic stenosis. The rupture of unstable plaques leads to coronary stenosis and acute coronary syndrome. Although calcified plaque has been considered as the established, stable and quiescent atheroma [26], recent studies reported that spotty distribution of calcium in calcified plaque was an important characteristic of vulnerable plaque leading to plaque rupture, which was more frequently observed in the culprit lesions of patients with acute coronary syndrome [27, 28]. Previously, a positive association between recurrent cardiovascular disease and high FGF23 levels was reported in The Heart Soul Study [12]. FGF23 was also positively associated with risk of incident heart failure (HF) and total cardiovascular events in the Cardiovascular Health Study [29] and with cardiovascular mortality in the Uppsala Longitudinal Study of Adult Men [30]. Aoki et al., however, reported that vascular calcification is not linked to other bone-related humoral factors including, FGF23, while serum FGF23 was elevated only in subjects with stage 4 nephropathy [31]. In another study, plasma cFGF23 levels did not significantly differ between diabetic patients and non-diabetic subjects [32].

Previous clinical studies have shown elevated OPG levels in patients with atherosclerosis and increased OPG levels with the severity of cardiovascular disease such as peripheral artery disease [33] and heart failure [34], with symptomatic carotid stenosis [35], unstable angina [36] and vulnerable carotid plaques [37]. The high concentrations of OPG could be responsible for a number of changes within the atherosclerotic plaque that would promote plaque instability. Within bone, OPG has been shown to modulate the release of matrix-degrading enzymes, such as cathepsins and therefore, it may also have an important influence on plaque stability [38].

In our population, we found that FGF23 serum levels were significantly increased and associated with USP independent of serum phosphorus level, which still remained within the normal range in the majority of the patients. Indeed, the increase in FGF23 concentration could be a compensatory mechanism to maintain serum phosphorus level in the normal range [39].

Our study has some potential limitations. It was a case–control study. Therefore, a recruitment and survival bias cannot be excluded. Our data were obtained from a cohort of European descent and includes subjects with other cardiovascular diseases. Thus, comorbidity might represent a confounding factor and the generalization of our findings to other age groups or ethnicities is unclear. The size of the population studied is relatively small and could lead to false positive results. Our findings need to be confirmed in larger samples and should also be tested in groups of different ethnic origins. We cannot rule out the possibility of residual confounding by other factors such as bone status, 1,25(OH)_2_D or PTH levels. Finally, prescribed or self-administered calcium and vitamin D supplements and dietary phosphate intake have not been considered in our calculations.

In conclusion, the present study identifies FGF23 serum levels as an independent risk factor for unstable carotid plaque in the general population with type 2 diabetes. The association was independent of traditional cardiovascular risk factors and kidney function. The data further suggest a role for FGF23 as a reliable biomarker in atherosclerotic disease. Future studies are needed to investigate the potential biological role of FGF23 in the pathogenesis of atherosclerotic disease and to evaluate whether FGF23 is a modifiable cardiovascular risk factor.

## Abbreviations

FGF23: Fibroblast growth factor 23
1,25(OH)_2_D: 1,25-dihydroxvitamin D
PTH: Parathyroid hormone
T2D: Type 2 diabetes
Hs-CRP: High-sensitivity C-reactive protein
IL-6: Interleukin-6
OPG: Osteoprotegerin
BMI: Body mass index
eGFR: Estimated glomerular filtration rate
CAD: Coronary artery disease
HIS: History of ischemic stroke
PAD: Peripheral artery disease
USP: Unstable plaque
SP: Stable plaque
HF: Heart failure

## References

[1] Shinohara Y, Nagayama M, Origasa H (2009). Postpublication external review of the Japanese guidelines for the management of stroke 2004. Stroke.

[2] Shimada T, Kakitani M, Yamazaki Y, Hasegawa H, Takeuchi Y, Fujita T (2004). Targeted ablation of Fgf23 demonstrates an essential physiological role of FGF23 in phosphate and vitamin D metabolism. J Clin Invest.

[3] Shimada T, Hasegawa H, Yamazaki Y, Muto T, Hino R, Takeuchi Y (2004). FGF-23 is a potent regulator of vitamin D metabolism and phosphate homeostasis. J Bone Miner Res.

[4] Wolf M (2012). Update on fibroblast growth factor 23 in chronic kidney disease. Kidney Int.

[5] Heine GH, Seiler S, Fliser D (2012). FGF-23: the rise of a novel cardiovascular risk marker in CKD. Nephrol Dial Transpl.

[6] Gutierrez OM, Mannstadt M, Isakova T, Rauh-Hain JA, Tamez H, Shah A (2008). Fibroblast growth factor 23 and mortality among patients undergoing hemodialysis. N Engl J Med.

[7] Jean G, Terrat JC, Vanel T, Hurot JM, Lorriaux C, Mayor B (2009). High levels of serum fibroblast growth factor (FGF)-23 are associated with increased mortality in long haemodialysis patients. Nephrol Dial Transpl.

[8] Mirza MA, Hansen T, Johansson L, Ahlstro¨m H, Larsson A, Lind L (2009). Relationship between circulating FGF23 and total body atherosclerosis in the community. Nephrol Dial Transpl.

[9] Mirza MA, Larsson A, Lind L, Larsson TE (2009). Circulating fibroblast growth factor-23 is associated with vascular dysfunction in the community. Atherosclerosis.

[10] Hu X, Ma X, Pan X, Hao Y, Luo Y, Lu Z (2015). Fibroblast growth factor 23 is associated with the presence of coronary artery disease and the number of stenotic vessels. Clin Exp Pharmacol Physiol.

[11] Roos M, Lutz J, Salmhofer H, Luppa P, Knauss A, Braun S (2008). Relation between plasma fibroblast growth factor-23, serum fetuin-A levels and coronary artery calcification evaluated by multislice computed tomography in patients with normal kidney function. Clin Endocrinol.

[12] Parker BD, Schurgers LJ, Brandenburg VM, Christenson RH, Vermeer C, Ketteler M (2010). The associations of fibroblast growth factor 23 and uncarboxylated matrix Gla protein with mortality in coronary artery disease: the heart and soul study. Ann Intern Med.

[13] Canziani ME, Tomiyama C, Higa A, Draibe SA, Carvalho AB (2011). Fibroblast growth factor 23 in chronic kidney disease: bridging the gap between bone mineral metabolism and left ventricular hypertrophy. Blood Purif.

[14] Dalal M, Sun K, Cappola AR, Ferrucci L, Crasto C, Fried LP (2011). Relationship of serum fibroblast growth factor 23 with cardiovascular disease in older community-dwelling women. Eur J Endocrinol.

[15] Parker BD, Schurgers LJ, Brandenburg VM, Christenson RH, Vermeer C, Ketteler M (2010). The associations of fibroblast growth factor 23 and uncarboxylated matrix Gla protein with mortality in coronary artery disease: the heart and soul study. Ann Intern Med.

[16] North American Symptomatic Carotid Endarterectomy Trial Collaborators (2011). Beneficial effect of carotid endarterectomy in symptomatic patients with high-grade carotid stenosis. N Engl J Med.

[17] Moore WS, Barnett HJ, Beebe HG, Bernstein EF, Brener BJ, Brott T (1995). Guidelines for carotid endarterectomy: a multidisciplinary consensus statement from the ad hoc committee, American heart association. Stroke.

[18] Executive Committee for the Asymptomatic Carotid Atherosclerosis (1995). Study endarterectomy for asymptomatic carotid artery stenosis. JAMA.

[19] Stary HC, Chandler AB, Dinsmore RE, Fuster V, Glagov S, Insull W (1995). A definition of advanced types of atherosclerotic lesions and a histological classification of atherosclerotic. A report from the committee on vascular lesions of council on atherosclerosis, american heart association. Circulation.

[20] Virmani R, Kolodgie FD, Burke AP, Farb A, Schwart SM (2000). Lessons from sudden coronary death: a comprehensive morphological classification scheme for atherosclerotic lesions. Arterioscler Thromb Vasc Biol.

[21] Straface G, Biscetti F, Pitocco D, Bertoletti G, Misuraca M, Vincenzoni C (2011). Assessment of the genetic effects of polymorphisms in the osteoprotegerin gene, TNFRSF11B, on serum osteoprotegerin levels and carotid plaque vulnerability. Stroke.

[22] Frink RJ, Achor RW, Brown AL, Kincaid OW, Brandenburg RO (1970). Significance of calcification of the coronary arteries. Am J Cardiol.

[23] Gutierrez OM, Januzzi JL, Isakova T, Laliberte K, Smith K, Callerone G (2009). Fibroblast growth factor 23 and left ventricular hypertrophy in chronic kidney disease. Circulation.

[24] Lim K, Lu TS, Molostvov G, Lee C, Lam FT, Zehnder D (2012). Vascular Klotho deficiency potentiates the development of human artery calcification and mediates resistance to fibroblast growth factor 23. Circulation.

[25] Xiao Y, Peng C, Huang W, Zhang J, Xia M, Zhang Y (2013). Circulating fibroblast growth factor 23 is associated with angiographic severity and extent of coronary artery disease. PLoS One.

[26] Abedin M, Tintut Y, Demer LL (2004). Vascular calcification: mechanisms and clinical ramifications. Arterioscler Thromb Vasc Biol.

[27] Ehara S, Kobayashi Y, Yoshiyama M, Shimada K, Shimada Y, Fukuda D (2004). Spotty calcification typifies the culprit plaque in patients with acute myocardial infarction: an intravascular ultrasound study. Circulation.

[28] Kataoka Y, Wolski K, Uno K, Puri R, Tuzcu EM, Nissen SE (2012). Spotty calcification as a marker of accelerated progression of coronary atherosclerosis: insights from serial intravascular ultrasound. J Am Coll Cardiol.

[29] Ix JH, Katz R, Kestenbaum BR, de Boer IH, Chonchol M, Mukamal KJ (2012). Fibroblast growth factor-23 and death, heart failure, and cardiovascular events in community-living individuals: CHS (cardiovascular health study). J Am Coll Cardiol.

[30] Arnlov J, Carlsson AC, Sundstrom J, Ingelsson E, Larsson A, Lind L (2013). Higher fibroblast growth factor-23 increases the risk of all-cause and cardiovascular mortality in the community. Kidney Int.

[31] Aoki A, Murata M, Asano T, Ikoma A, Sasaki M, Saito T (2013). Association of serum osteoprotegerin with vascular calcification in patients with type 2 diabetes. Cardiovasc Diabetol.

[32] van Ark J, Hammes HP, Van Dijk MC, Lexis CP, van der Horst IC, Zeebregts CJ (2013). Circulating alpha-klotho levels are not disturbed in patients with type 2 diabetes with and without macrovascular disease in the absence of nephropathy. Cardiovasc Diabetol.

[33] Ziegler S, Kudlacek S, Luger A, Minar E (2005). Osteoprotegerin plasma concentrations correlate with severity of peripheral artery disease. Atherosclerosis.

[34] Ueland T, Yndestad A, Øie E, Florholmen G, Halvorsen B, Frøland SS (2005). Dysregulated osteoprotegerin/RANK ligand/RANK axis in clinical and experimental heart failure. Circulation.

[35] Golledge J, McCann M, Mangan S, Lam A, Karan M (2004). Osteoprotegerin and osteopontin are expressed at high concentrations within symptomatic carotid atherosclerosis. Stroke.

[36] Sandberg WJ, Yndestad A, Øie E, Smith C, Ueland T, Ovchinnikova O (2006). Enhanced T cell expression of RANK ligand in acute coronary syndrome: possible role in plaque destabilization. Arterioscler Thromb Vasc Biol.

[37] Kadoglou NP, Gerasimidis T, Golemati S, Kapelouzou A, Karayannacos PE, Liapis CD (2008). The relationship between serum levels of vascular calcification inhibitors and carotid plaque vulnerability. J Vasc Surg.

[38] Wittrant Y, Couillaud S, Theoleyre S, Dunstan C, Heymann D, Redini F (2002). Osteoprotegerin differentially regulates protease expression in osteoclast cultures. Biochem Biophys Res Commun.

[39] Fliser D, Kollerits B, Neyer U, Ankerst DP, Lhotta K, Lingenhel A (2007). Fibroblast growth factor 23 (FGF23) predicts progression of chronic kidney disease: the mild to moderate kidney disease (MMKD) study. J Am Soc Nephrol.

